# The Effects of a Passive Exoskeleton on Human Thermal Responses in Temperate and Cold Environments

**DOI:** 10.3390/ijerph18083889

**Published:** 2021-04-08

**Authors:** Yang Liu, Xiaoling Li, Jiarui Lai, Aibin Zhu, Xiaodong Zhang, Ziming Zheng, Huijin Zhu, Yueyang Shi, Long Wang, Zhangyi Chen

**Affiliations:** School of Mechanical Engineering, Xi’an Jiaotong University, Xi’an 710000, China; xjtu_liuyang@stu.xjtu.edu.cn (Y.L.); ljr285@stu.xjtu.edu.cn (J.L.); abzhu@mail.xjtu.edu.cn (A.Z.); amct@mail.xjtu.edu.cn (X.Z.); zmzheng121719@stu.xjtu.edu.cn (Z.Z.); zhhjjym@stu.xjtu.edu.cn (H.Z.); victories@stu.xjtu.edu.cn (Y.S.); wangl521@stu.xjtu.edu.cn (L.W.); 3120101240@stu.xjtu.edu.en (Z.C.)

**Keywords:** exoskeleton, thermal response, metabolic heat production, thermal comfort, thermal sensation

## Abstract

The exoskeleton as functional wearable equipment has been increasingly used in working environments. However, the effects of wearing an exoskeleton on human thermal responses are still unknown. In this study, 10 male package handlers were exposed to 10 °C (COLD) and 25 °C (TEMP) ambient temperatures while performing a 10 kg lifting task (LIFTING) and sedentary (REST) both with (EXO) and without the exoskeleton (WEXO). Thermal responses, including the metabolic rate and mean skin temperature (MST), were continuously measured. Thermal comfort, thermal sensation and sweat feeling were also recorded. For LIFTING, metabolic heat production is significant decrease with the exoskeleton support. The MST and thermal sensation significantly increase when wearing the exoskeleton, but thermal discomfort and sweating are only aggravated in TEMP. For REST, MST and thermal sensation are also increased by the exoskeleton, and there is no significant difference in the metabolic rate between EXO and WEXO. The thermal comfort is significantly improved by wearing the exoskeleton only in COLD. The results suggest that the passive exoskeleton increases the local clothing insulation, and the way of wearing reduces the “pumping effect”, which makes a difference in the thermal response between COLD and TEMP. Designers need to develop appropriate usage strategies according to the operative temperature.

## 1. Introduction

Wearable equipment is increasingly used in daily life, and the exoskeleton is one of them. In manual work scenarios, workers are exposed to the risk of work-related musculoskeletal disorders (WMSDs). In the United States alone, the annual economic loss caused by WMSDs is as high as $50 billion USD [1]. Although automation and intelligent robots reduce the need for manual operations, due to the flexibility of manual operations, many manual tasks still require the experience and skills of workers. Due to the advantages of small size and wearability, the exoskeleton as a support device has attracted widespread attention and research in the industrial field in recent years. These exoskeletons can be used for maintenance, assembly or handling in logistics [2,3]. The power forms of the industry exoskeleton include passive and active forms. The passive exoskeleton does not require an external energy supply, which only relies on its mechanical damping to provide support for the workers, so it is low-cost and lighter than the active type. At present, passive exoskeletons are mostly used to support the upper limbs and spine during manual work.

Industrial exoskeletons innovatively provide supporting intervention from the work characteristics of workers to alleviate the workload. Existing studies of exoskeletons have shown that they can reduce muscle fatigue, perceived efforts, and thus have benefits for preventing WMSDs [4,5,6]. However, these advantages to the human body can easily make people ignore the impact of environmental factors on the ergonomics in exoskeleton application. Since working environments such as construction sites and warehouses are open-air or lack the capability of temperature adjustment, the application environment of the industrial exoskeleton is complicated. Some automobile factories have observed that it is difficult to control the workshop temperature below 25 °C even with cooling equipment in hot weather, which greatly affects production efficiency [7,8]. In high latitude areas, cold exposure is a common problem faced by work [9]. In a recent report, workers were asked to use a supporting exoskeleton for overhead assembly in the automotive manufacturing factory, and the questionnaire found that thermal discomfort becomes a major factor in disuse. Workers requested to improve the thermal comfort of the exoskeleton for long-term use [2]. ASHRAE Standard 55 stipulates the indoor acceptable operative temperature with the applicable prerequisites of the physical activity (1.0 to 1.3 met, 58 W/m^2^ metabolic rate converted to 1 met) [10]. However, the level of physical activity is higher in the exoskeleton usage scenario, and people working in the open air are often exposed to hot or cold environments.

Workers and the environment carry out continuous heat exchange to maintain the core body temperature of the human body. Changes in the environmental temperature affect the human body’s thermal response, including physiology and psychology. The ASHRAE uses the scales to quantify people’s thermal sensation and thermal comfort [10]. When the human body feels hot, the thermal sensation deviates from “neutral” and shifts to the “hot” direction. At the same time, the acute circulatory system response of the human body increases the heart rate, blood flow and sweating [11]. Cardiovascular activity promotes blood flow to the surface of the body to increase peripheral blood volume. When the ambient temperature is overheated or physical activity increases, sweat evaporates at the ambient temperature to reduce the skin temperature [12,13]. In the study of physiological parameters and subjective responses, it was found that there is a strong correlation between skin temperature and thermal sensation [14]. Therefore, the mean skin temperature (MST) calculated by local skin temperatures and the corresponding weighting factors is usually measured as a physiological parameter related to thermal comfort [15,16]. Contrary to a hot environment, when the human body is exposed to a cold environment, the heat transfer from the core of the body to the shell. The contraction of blood vessels enhances the insulation capacity of the skin and subcutaneous tissues [17,18]. When the heat dissipation of the human body in a cold environment makes it difficult to maintain the core temperature, the sympathetic nervous system becomes active and increases the metabolic rate of cells to generate heat [19]. The stimulation of β-adrenergic receptors by the sympathetic nervous system activates brown adipose tissue (BAT), resulting in metabolic heat production. The activity level also affects the thermal response. In previous studies, under a higher metabolic rate condition (e.g., mechanical work), the metabolic rate has a stronger effect on thermal comfort than ambient temperature [20].

Occupational heat exposure affects work productivity, physical and cognitive ability and even brings safety risks to workers [21,22]. The increase in physical activity makes the metabolic activity more active and increases the heat sensation and sweating rate, which further increases the physical stress of workers [23,24,25]. The factory can alleviate the negative impact of heat exposure on workers by reducing the work intensity and optimizing the work–rest schedule [26]. Previous studies have shown that exoskeletons have positive results in the biomechanical evaluation. It has been proven to significantly reduce the muscle activity of the main muscles involved in handling or overhead work [27,28]. The passive back exoskeleton has been shown to reduce spinal muscle activity by 20 to 25% in real car assembly operations [29]. In a comparative experiment involving eight workers, a passive assisted exoskeleton significantly reduced the back muscle activity under the repeated lifting task [30]. When using the upper limbs exoskeleton in the overhead work, the muscle activity of the biceps brachii and the medial deltoid muscle was reduced by 49% and 41%, respectively [31]. However, contrary to our current knowledge, the subjective feeling of thermal discomfort is strong when the exoskeleton reduces muscle activity.

Cold exposure would cause impairments in human physiological functions, especially muscular functioning. The accuracy of the worker’s operation decreases under the cold operation, largely due to the decrease of the hand temperature [32,33]. In general, people in cold environments need protective measures to improve thermal comfort [34]. In standing and assembly tasks, wearable personal heating devices for limbs and torso have been proven to significantly improve the thermal comfort of workers [35]. The required clothing insulation during the light physical activity under 4 to 10 °C is 1.6 to 1.9 CLO [36]. However, with the increase in activity level, the body’s thermal sensation can be significantly improved. When the activity level is high, it may cause increased sweating, and people will take the initiative to reduce clothing for heat dissipation. In a cold environment, the risk of hyperthermia is possible when working with protective clothing [37]. Although changes in activity status make the thermal response show different results in a cold environment, the effect of the exoskeleton’s activity intervention on the thermal response is still unclear.

A key feature of the exoskeleton is wearability, which forms a dynamic structure with the human body’s movement joints. The passive upper or lower limbs exoskeleton mainly forms contact with the chest, trunk, thighs, shoulders and upper arms to support and reduce joint torque under static or dynamic operations. The material used in the exoskeleton is different from that of common clothing. The outer layer of the exoskeleton can be made of ABS plastic, carbon fiber or alloy materials [38,39,40]. The inner side in contact with the human body uses memory foam, interlining, and mesh to prevent the high-strength surface material from harming the workers [41]. Organic polymer materials are widely used in the clothing industry, and the combination of inorganic polymer materials and fabrics is reported to have better thermal insulation properties [42,43]. Cold protective clothing with the metal coating is reported to increase the thermal resistance of fabrics by 30 to 75% [44]. The mixed structure of multiple materials used in the exoskeleton may potentially increase the clothing insulation, which may also affect the thermal response of workers.

Unlike other wearable devices, the exoskeleton can cause changes in muscle activity, but its influence on the thermal response of the human body is unknown. This paper aims to study the effects of a passive exoskeleton on the thermal response of the human body. The exoskeleton is mainly used to reduce the lower back load of the human body during repeated operations. Although previous studies have shown that exoskeletons can provide good assistance, they did not explain the impact of assistance on human thermal response and report the temperature under the experiment. Metabolic heat production is closely related to the activity level, and it is also meaningful to compare the thermoregulatory activity of workers using it under different temperature. This study analyzes other thermal responses, including the mean skin temperature, thermal sensation, and thermal comfort, which helps to evaluate wearability under environmental variables. To the best of our knowledge, this is the first study to investigate the thermal response when wearing the exoskeleton under two temperatures. We hope to explore whether the exoskeleton’s effect on human thermal comfort will become a potential problem in its promotion and application through this research.

## 2. Materials and Methods

### 2.1. Participants

Ten healthy male package handlers who work for a Chinese courier company were recruited for this experiment. They all had more than one year of work experience and no history of musculoskeletal diseases. To ensure the validity of experimental data, the subjects were reminded to avoid caffeine and alcohol ingestions, smoking and strenuous activities, and sleep well the day before the experiment. The mean values (standard deviation, SD) of age, height, and body mass were 24.4 (2.7) years, 1.72 (5.14) cm, and 66.0 (11.3) kg, respectively. Before the experiment, subjects chose a clothing insulation between 0.5 to 1.3 CLO according to their occupational habits. For unified measurement standards, all subjects were required to wear long underpants, straight trousers, a long-sleeved sweatshirt, socks, boots, and a jacket. The total insulation value calculated was approximately 1.27 CLO [10].

This study was approved by the Institutional Review Board at Xi’an Jiaotong University and was performed following the ethical standards of the 1964 Declaration of Helsinki (2008). Every subject gave their written informed consent to participate.

### 2.2. Passive Exoskeleton

**Figure 1:**
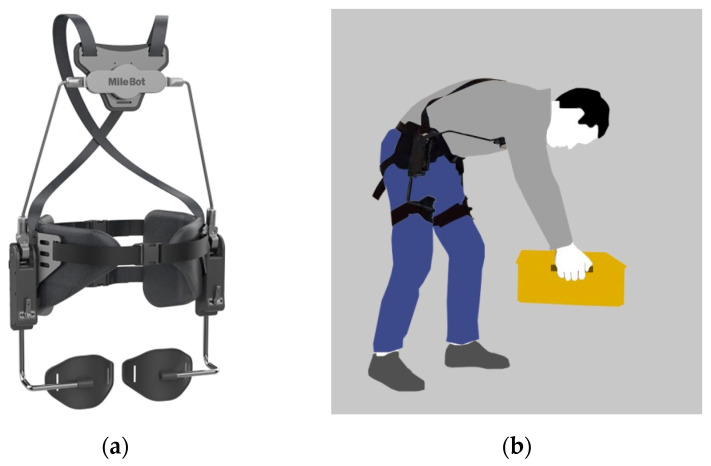
The passive exoskeleton. (**a**) Structure of the passive exoskeleton; (**b**) human–machine interaction of the passive exoskeleton.

In the study, a passive exoskeleton (Mile Bot, Shenzhen, China) was used, as presented in Figure 1a. This exoskeleton is designed to protect high-frequency repetitive bending workers. It includes a spacer for the chest and two spacers for the thighs. The two parts are connected by a supporting structure containing a damping mechanism that provides support at the hip joints. The exoskeleton is adjusted and fixed to the body by straps on the back and crotch. During work, the upper body of the human body is supported by the spacers on the chest and thighs, and the damping mechanism in the middle provides the support force, thereby reducing the muscle activity of the lower back (Figure 1b). The weight of this exoskeleton is 3.0 kg, and the maximum support torque is 38 N/m.

### 2.3. Experimental Design

This paper employed a repeated-measures design to study the effects of the passive exoskeleton on the human thermal response. The independent variables of two temperature conditions are 25 °C (TEMP) and 10 °C (COLD), which are selected according to China’s hot summer–cold winter climate region [45]. The operating states were wearing (EXO) and without an exoskeleton (WEXO). The dependent variables are the physiological responses and subjective responses, including the metabolic rate, mean skin temperature, thermal sensation, thermal comfort, and perceived sweating index.

**Figure 2:**
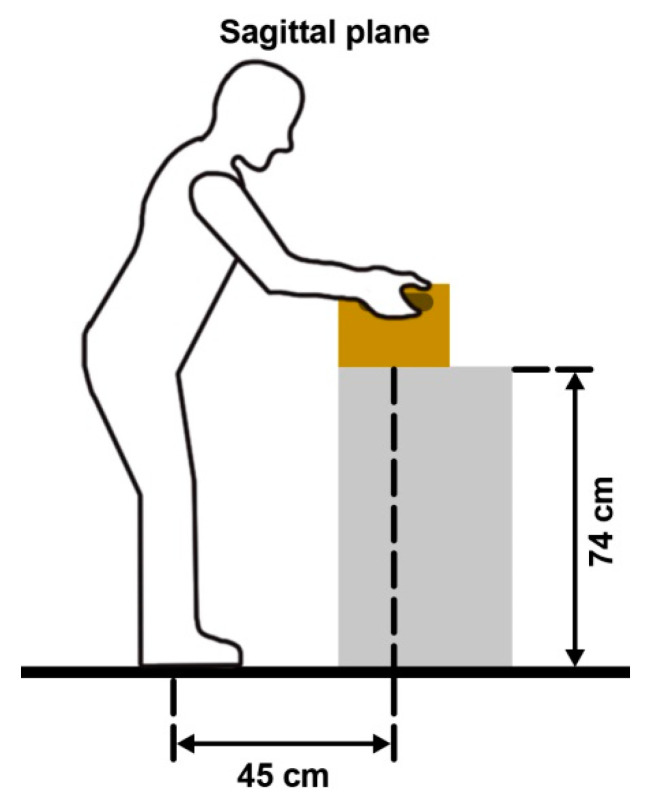
LIFTING task according to the NIOSH Lifting Equation.

Since workers are not always working, we designed two activity states: REST and LIFTING. Under REST, subjects were required to sit in a chair to rest. For LIFTING, in order to ensure the safety of the subjects, the lifting load and height were designed according to the National Institute of Occupational Safety and Health (NIOSH) Lifting Equation (Figure 2) [46]. The subjects were asked to operate a lift and lower task with a 10 kg box to and from a 74 cm high platform. The size of the box was set as 32.7 (length) × 23.6 (width) × 24 (height) cm, which was placed 45 cm in front of the subject’s prescribed standing position. The order of the experiment was randomly assigned by a computer. Each participant performed all the activity states and temperature conditions under both EXO and WEXO. The interval between each experiment of the subjects was five days.

### 2.4. Procedure

Subjects were asked to enter the experiment two hours after eating to eliminate the effects of digestion and metabolism. Subjects arrived in the preparation room 40 min before the start of the experiment. During preparation, a researcher recorded anthropometric data and informed the subjects of the procedure and the equipment involved in the experiment. Then, the facilitator helped the subjects wear and adjust the physiological data collection equipment and the exoskeleton. To avoid the interference of the exoskeleton, the collection devices were placed in a position where it did not interfere with the exoskeleton. Then, they entered a chamber and were sedentary (26 °C, 50% relative humidity, air velocity <0.1 m/s) for a 20-min adaptation period.

**Figure 3:**
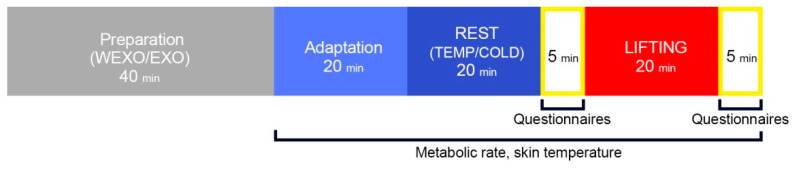
Experimental procedure.

Once completed, subjects entered another chamber that had a pre-determined temperature. They sat on a chair for REST. When resting with the exoskeleton, the damping mechanisms at the hip are disconnected from cancelling the support. After the REST, the participant was directed to the designated location for LIFTING so that their bodies were parallel to the platform on the coronal plane. In order to obtain constant activity, the whole process was guided by a metronome. When the metronome sounded, the participant lifted the box to the table, and when the beat sounded again, it would be placed on the floor from the table. This cycle was performed five times per minute. In this process, the subjects could choose their comfortable lifting technique. Figure 3 shows the experimental procedure of this study. The environmental parameters tested in the experiments were relatively stable within the ranges summarized in Table 1.

According to a previous study, the metabolic rate increased progressively in the first 2 to 3 min of exercise, and then the steady-state metabolic rate was obtained from 3 to 8 min [47]. Subjective thermal sensation and skin temperature tend to reach a steady state after 15 min of activity [48]. In this study, the subjects were asked to maintain 20 min for REST and LIFTING, then fill out the questionnaires within 5 min. The skin temperature and metabolic rate were continuously collected. We used the last 5 min of metabolic rate and skin temperature as the steady-state physiological responses.

### 2.5. Physiological and Subjective Measurements

#### 2.5.1. Metabolic Rate

An automated breath-by-breath system (METAMAX metabolic device, CORTEX, Germany) was used to continuously measure the volumetric rate of oxygen consumption and carbon dioxide production using a breathing mask with a Nafion filter tube and a turbine flow meter. Then, the metabolic rate was calculated by the following equation [49]:

(1) $$M=\frac{21\left(0.23RQ+0.77\right)QO_{2}}{A_{d}}$$

where M is the metabolic rate (W/m^2^). *QCO*_2_ is the volumetric rate of carbon dioxide production and *QO*_2_ is the volumetric rate of oxygen consumption. *RQ* is the respiratory quotient, which is the molar ratio of *QCO*_2_ (L/min) exhaled to *QO*_2_ (L/min) inhaled, and *A_d_* is the Dubois surface area (m^2^). It can be determined by following the empirical equation [50]:

(2) $$A_{d}=0.202H^{0.725}W^{0.425}$$

where *H* is height (m) and *W* is weight (kg).

#### 2.5.2. Mean Skin Temperature (MST)

The region skin temperature was recorded using the iButton (Maxim Integrated, CA, USA), placed on the forehead, chest, upper arm, forearm, hand, anterior thigh, anterior calf and foot of the right side of the body. The skin temperature of each site was recorded every 1 min throughout the experimental session. The MST was calculated using Gagge/Nishi’s equation [51]:

(3) $$T_{MSK}=0.07T_{forehead}+0.175T_{chest}+0.175T_{upperarm}+0.07T_{forearm}+0.07T_{hand}+0.05T_{calf}+0.19T_{thigh}+0.2T_{foot}$$

#### 2.5.3. Questionnaires Survey

**Figure 4:**
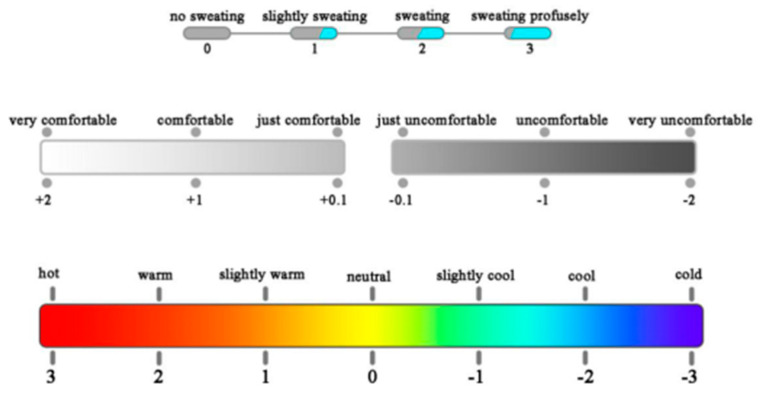
Scales for SFI, TCV, and TSV.

As shown in Figure 4, the thermal sensation vote (TSV) was divided into ASHRAE seven-point scales: hot (+3), warm (+2), slightly warm (+1), neutral (0), slightly cool (−1), cool (−2), and cold (−3). The thermal comfort vote (TCV) was divided into six scales: very comfortable (+2), comfortable (+1), just comfortable (+0.1), just uncomfortable (−0.1), uncomfortable (−1), and very uncomfortable (−2) [10]. The sweat rate is one of the significant characteristics of body temperature regulation. People can judge whether they sweat and how strongly they are sweating by using the sweat feeling index (SFI) to investigate the influence of sweat activity on skin temperature and thermal sensation. SFI is scaled as 0, 1, 2, and 3, which indicates that subjects have no feeling of sweating, a slight feeling of sweating, a moderate feeling of sweating, and a strong feeling of sweating, respectively.

#### 2.5.4. Statistical Method

Three factors, repeated-measures ANOVA, were performed to compare each of the dependent variables (metabolic rate, MST, TSV, TCV and SFI) from two temperature conditions both with and without the exoskeleton. All data were assessed for the approximation to a normal distribution and sphericity, and when necessary, degrees of freedom were adjusted using the Greenhouse–Geisser adjustment. When an ANOVA revealed significance, Bonferroni’s *t*-test was conducted in the further post hoc test. The statistical analysis was performed using SPSS statistical software (V22, Chicago, IL, USA). When the test result showed significance of difference, it was labeled as * *p* < 0.05, ** *p* < 0.01, *** *p* < 0.001. All data (Appendix A) are reported as mean (SD).

## 3. Results

### 3.1. Results of Metabolic Rate

**Figure 5:**
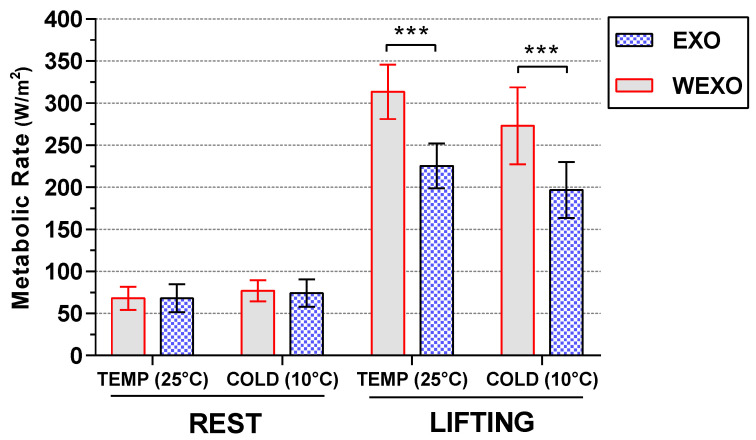
Metabolic rate in steady-state condition (*** *p* < 0.001).

The results of the metabolic rate are shown in Figure 5. There was a main effect on the metabolic rate for activity (F (1, 9) = 586.83, *p* < 0.001) and temperature (F (1, 9) = 18.48, *p* = 0.003). Across two exoskeleton conditions, the metabolic rate in LIFT was dramatically higher than REST. Repeated measures ANOVA also revealed an effect on metabolic rate for activity with the exoskeleton (F (1, 9) = 210.76, *p* < 0.001). For LIFT, pairwise comparisons demonstrated that the exoskeletal intervention decreased the metabolic rate in the TEMP from 313.49 (SD = 32.31) W/m^2^ to 225.53 (SD = 26.53) W/m^2^ (F (1, 9) = 126.48, *p* < 0.001), and in the COLD, from 273.15 (SD = 45.80) W/m^2^ to 196.73 (SD = 33.39) W/m^2^ (F (1, 9) = 51.05, *p* < 0.001). The result shows a slight increase under REST between WEXO and EXO in both temperature conditions but with no significant difference between them. LIFT in the TEMP environment was significantly higher than the same task condition in the COLD environment (F (1, 9) = 25.18, *p* = 0.001).

### 3.2. Results of MST

**Figure 6:**
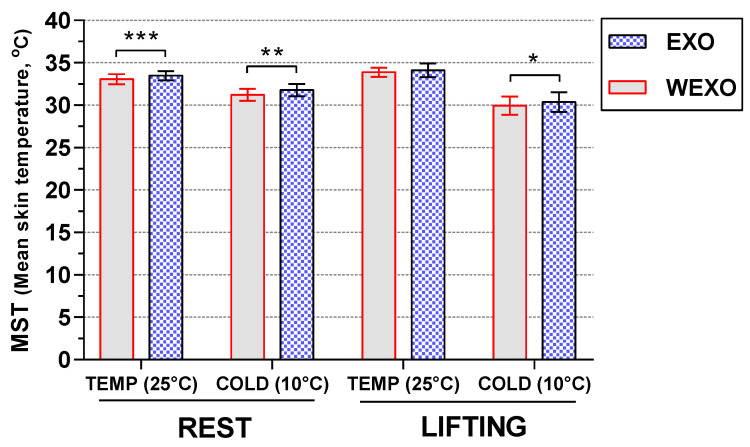
MST in steady−state condition.3.3. Results of TSV (* *p* < 0.05, ** *p* < 0.01, *** *p* < 0.001).

Results of MST are shown in Figure 6. Repeated measures ANOVA revealed a significant main effect for the temperature (F (1, 9) = 117.99, *p* < 0.001) and exoskeleton (F (1, 9) = 61.66, *p* < 0.001). The MST in TEMP was higher than COLD under each experimental session. The MST in EXO was higher than WEXO both in TEMP and COLD. Post hoc *t*-tests revealed that for the REST, the MST under EXO increased by 0.41 (F(1, 9) = 64.96, *p* < 0.001) and 0.56 (F(1, 9) = 20.30, *p* = 0.001) in TEMP and COLD, respectively. For the LIFT, the MST under EXO increased by 0.25 (F(1, 9) = 2.71, *p* > 0.05) and 0.42 (F(1, 9) = 9.89, *p* < 0.05) in TEMP and COLD, respectively. A significant temperature × activity interaction effect was also observed (F (1, 9) = 94.01, *p* < 0.001), where MST under LIFT was 0.73 °C higher than REST in TEMP (F (1, 9) = 29.19, *p* < 0.001). However, there was a 1.34 °C decrease between REST and LIFT in COLD (F (1, 9) = 41.34, *p* < 0.001).

### 3.3. Results of TSV

**Figure 7:**
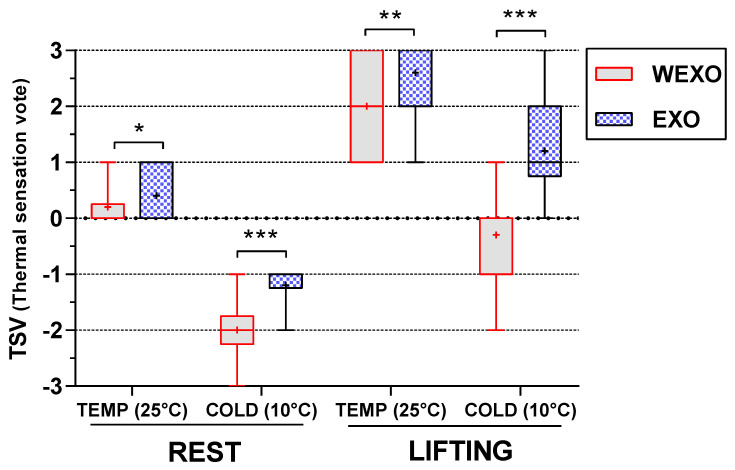
Thermal sensation vote in steady−state condition (* *p* < 0.05, ** *p* < 0.01, *** *p* < 0.001).

The results of TSV can be seen in Figure 7. The results found a significant effect for exoskeleton (F (1, 9) = 56.54, *p* < 0.001) and exoskeleton× temperature interaction (F (1, 9) = 36.00, *p* < 0.001). Post hoc *t*-tests revealed that TSV was significantly improved under each EXO sessions. For the TEMP, the TSV under REST and LIFT was increased by 0.17 and 0.16, respectively, with EXO as compared to WEXO. Furthermore, the effect of the exoskeleton was more pronounced for the COLD, where TSV under REST and LIFT were increased by 0.80 and 1.50, respectively. Repeated measures ANOVA also revealed a main effect on TSV for temperature (F (1, 9) = 213.89, *p* < 0.001) and task (F (1, 9) = 110.40, *p* < 0.001), where TSV significantly increased with the increase in temperature and task intensity.

### 3.4. Results of TCV

**Figure 8:**
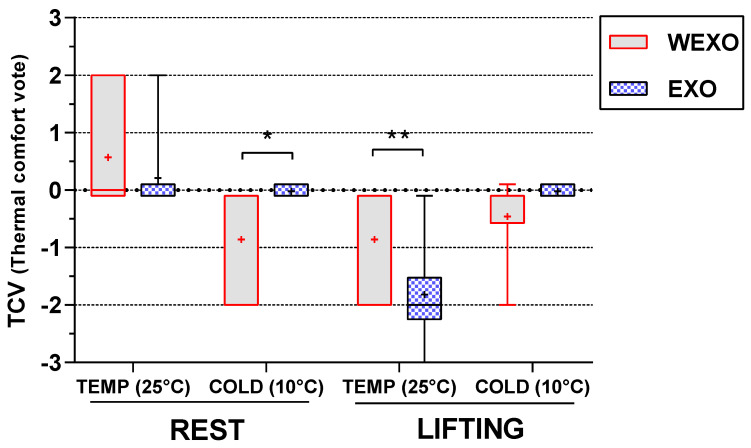
Thermal comfort vote in steady−state condition (* *p* < 0.05, ** *p* < 0.01).

Figure 8 shows the result of the TCV. A significant exoskeleton × temperature interaction (F (1, 9) = 83.12, *p* < 0.001) was observed in the results of the repeated measures ANOVA. Further analysis found thermal comfort with a 0.96 deterioration between WEXO and EXO during LIFT in TEMP (F (1, 9) = 11.48, *p* = 0.008) and a 0.84 improvement between WEXO and EXO during REST in COLD (F (1, 9) = 7.65, *p* < 0.05). There was also a significant main effect for activity × temperature interaction (F (1, 9) = 37.50, *p* < 0.001), which was driven by a significant increase of the TCV in REST from COLD to TEMP (F (1, 9) = 8.195, *p* < 0.05), but a significant decrease during LIFT from COLD to TEMP (F (1, 9) = 21.04, *p* = 0.001).

### 3.5. Results of SFI

**Figure 9:**
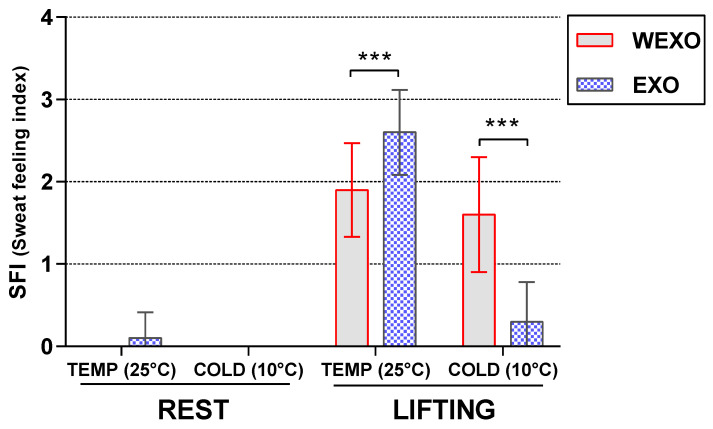
Sweat feeling index under WEXO and EXO (*** *p* < 0.001).

Result for SFI (Figure 9) demonstrated a significant main effect for task (F (1, 9) = 146.59, *p* < 0.001). Post hoc *t*-tests revealed that SFI increased between LIFT and REST in both temperature and exoskeleton conditions. Exoskeleton × temperature interaction (F (1, 9) = 30.78, *p* < 0.001) was observed in the results. Further analysis in LIFT found a significant increase between WEXO and EXO in TEMP (F (1, 9) = 96.00, *p* < 0.001). Conversely, SFI significantly decreased in the COLD under the same task and exoskeleton conditions (F (1, 9) = 81.00, *p* < 0.001).

## 4. Discussion

### 4.1. Effects of Wearing Exoskeleton on Thermal Responses under LIFTING

Our results showed that this passive exoskeleton led to a significant reduction of metabolic rate during lifting task in both TEMP and COLD. Metabolic rate is used for measuring activity levels in ASHRAE Standard 55. The decrease in the metabolic rate indicates that the use of the exoskeleton reduces the work intensity and can play an effective role in working support. As the lower back muscles need to bear a relatively large load during lifting work, shear forces are formed at the L5/S1 joints [52]. The exoskeleton used in this study is designed to provide support for the lower back. The support can reduce the peak and cumulative load on the L5/S1 joints, thereby reducing the metabolic rate in work. As the TSV in the hot environment deviates more from the neutral thermal sensation than in the cold environment, we also find that LIFTING in the TEMP environment had a higher metabolic rate than in the COLD environment, which still exists under EXO. A closer to neutral thermal sensation corresponds to a lower metabolic rate, while the bias towards the “cold” or “hot” thermal sensation corresponds to a higher metabolic rate [50].

Under WEXO, we observed that repetitive work caused a high activity level (5.4 met in TEMP and 4.7 met in COLD). Heat production under the high metabolic rate condition needs to continuously dissipate to maintain the core temperature and promote thermal comfort. Similar to some studies on thermoregulatory behavior during continuous exercise [13,53], we observed higher sweating and thermal discomfort under LIFTING. We found that the metabolic rate under EXO has been greatly reduced, and the activity level has dropped to 3.9 met and 3.4 met in TEMP and COLD, respectively. The degree of environmental improvement (including a reduction in the preferred ambient temperature and a wind speed increase) to maintain human thermal comfort will decrease as activity intensity decreases [54], which indicates that thermal comfort can be achieved by reducing the intensity of the activity while the environmental variables remain unchanged. We have only observed the improvement of thermal comfort after using the exoskeleton in the COLD environment. However, the support effect of using an exoskeleton under TEMP does not make up for the improvement of thermal comfort caused by insufficient regulation of environmental factors. Previous studies have observed that low activity levels in a warm environment have better thermal comfort than high activity levels [16,54], which shows the different results from our research. The passive activity regulation caused by the exoskeleton and the spontaneous activity regulation of the human body may have different physical and psychological thermal response mechanisms. The metabolic rate change caused by the activity level has a significant impact on thermal comfort prediction. When the MET exceeds 1.8 MET, it will cause a calculation error of 1 unit of PMV. Although many studies have modified the PMV model to varying degrees to make it suitable for predicting thermal comfort at high activity levels, the mechanism of thermoregulation under the activity intervention of wearable devices still needs further research. In addition to the metabolic rate, clothing insulation is also one of the six primary factors when it defines the condition of thermal comfort [10]. The wearability of the exoskeleton is similar to the clothing, which means that although the exoskeleton reduces the activity intensity, the added clothing insulation improves the metabolic heat production, and the human body increases sweating to maintain the thermal balance. In addition to the clothing insulation, it is also necessary to pay attention to the way of wearing the exoskeleton. As the stability of the force transmission needs to be considered in the design to produce a better support effect, the exoskeleton needs to be tightly attached to the human body through the strap or the fixing device. In this study, the place where the exoskeleton is in contact with the human chest, back, crotch, and thighs are designed to be worn with a binding method, which causes the reduction of the “pumping effect” during work. The pumping effect means that the openings of the clothing allow the human–clothing space to conduct air exchange with the external environment, and this air exchange will increase during activity [55]. The weakening of the pumping effect reduces the evaporation and heat dissipation of sweat during the air exchange. This situation generally occurs when wearing protective clothing [56]. The difference is that the exoskeleton only increases the local clothing insulation while the protective clothing increases the area more. The same point is that they both hinder the pumping effect. Thermal discomfort was reported to be the main reason for not wearing the exoskeleton in a thermal environment or summer. After researchers improved materials and increased airflow channels for reducing thermal discomfort, workers still demanded to improve cool strategies further [2]. The effect of the way of wearing the exoskeleton on thermal comfort may be greater than the increase in inherent clothing insulation of the exoskeleton. However, this variable needs to be verified by further research.

Although we also observed an increase in skin temperature in the COLD environment, sweating is significantly reduced. Without the assistance of the exoskeleton, the human body sweats and dissipates heat in order to maintain thermal balance at high activity intensity. As excessive sweating will cause clothing to become wet, it will affect the warmth of the clothing [57], and the TSV on the cold side when subjects perform LIFTING in the COLD environment. Under the EXO, the increase in clothing insulation and the decrease in the pumping effect enhance the warmth retention effect of the clothes, which improves the human body’s thermal sensation in cold environments. Simultaneously, the reduction in sweating is also conducive to the improvement of thermal comfort [58,59]. Although the increase in TCV is not statistically significant, it is already very close to “thermal comfort”. People are more sensitive to cold than warmth and activity reduces the thermal sensation to hot stimulation [60,61] and the degree of increase of the thermal sensation in COLD environments more than in TEMP environments when using the exoskeleton. Previous studies reported the correlation between the mean skin temperature and manual task performance under cold exposure. The working ability decreases as the mean skin temperature decreases [62]. Therefore, the observed increase in skin temperature indicates that the exoskeleton improves work performance in cold environments.

### 4.2. Effects of Wearing an Exoskeleton on Thermal Responses under REST

Wearing an exoskeleton has no significant effect on the metabolic rate in a sitting environment, but the mean skin temperature and thermal sensation increase significantly. Luo et al. found that the change of clothing insulation from 0.42 to 0.91 CLO at 26 °C only increased the TSV but not the metabolic rate [50]. The change in clothing insulation seems to have less effect on metabolic rate in temperate environments. The metabolic rate is 68.08 W/m^2^ (about 1.2 MET) in this study, and the experimental environment of 25 °C meets the 90% operative temperature limits stipulated by ASHRAE Standard 55 for the average temperature of the month from 10 °C to 33 °C [10]. What caught our attention was that there is still a sweating report in the result, and the TCV also has a downward trend. In many areas, the outdoor temperature reported in summer has exceeded the maximum limit of 33 °C used by ASHRAE Standard 55. The temperature of the workshop may be higher due to poor air circulation. Therefore, wearing an exoskeleton to rest in these environments is more likely to cause thermal discomfort.

In the COLD environment, the study did not find a significant difference in the metabolic rate between with and without the exoskeleton. The same as the previous study is that the metabolic rate is higher than that in TEMP [19], but it is not significant. In this study, improvement of the TSV and TCV caused by a multi-point combination of increased local clothing insulation has not been reported in the previous research. Body regions have different effects on overall thermal sensation, with the back, chest and pelvis being the most influential parts [63]. The inner side of the crotch damping mechanism was added with a multi-layer material to relieve the friction between the exoskeleton and the human body, which greatly improved the clothing insulation on the pelvis. The clothing insulation on the chest was also improved by the support sheet of the exoskeleton. For these reasons, the TSV during a sedentary period is improved. The increased local clothing insulation also significantly increases the MST of the sedentary period in cold temperatures. The thermoregulatory and control of human skin blood flow is essential to maintain body temperature. Heat can be transported from the local to the whole body through blood circulation [64].

### 4.3. Limitations and Future Works

There are limitations to this work. We have studied the effect of the exoskeleton’s wearability on the thermal response of the human body but have not calculated the changes in clothing insulation after wearing the exoskeleton. In this study, the materials of the exoskeleton include ABS plastic, metal, sponge, and mesh cloth. At present, the more advanced auxiliary exoskeleton also uses carbon fiber to reduce weight. For the clothing insulation of the wearable equipment, we will use heat transfer simulation analysis to calculate it in further research. Moreover, the way of wearing an exoskeleton is also different from that of common clothing. Many existing calculation models of clothing insulation for movement may not be suitable for wearing the exoskeleton.

Personnel clothing has a strong correlation with the weather and indoor climate. In order to study the temperature and motion variables, this study sacrificed the effect of changes in clothing insulation on exoskeleton use. Clothing insulation variables should also be included in future research to better analyze the effect of wearing an exoskeleton on acceptable operative temperature.

As previous studies have reported, the changes in thermal sensation in local parts have different effects on the overall thermal sensation. The way of wearing the exoskeleton is designed according to the use scene and the support strategy. In addition to the passive exoskeleton used in this study for the lower-back assistant, there also are exoskeletons supporting the upper and lower limbs, and they should also be studied in the future to obtain comprehensive results for the application environment.

In addition, the number of subjects in this study is the same as those in references [5,27] and higher than those in some previous researches such as [28,30], but more subjects, including female subjects, should be involved in the experiment in order to obtain more reliable results in the future.

## 5. Conclusions

The aim of the present research was to evaluate the effect of exoskeleton use on human thermal responses. We experimentally evaluated the human thermal response with and without a passive exoskeleton under REST and LIFTING in two temperatures. The main conclusions are summarized below:

1. Compared to without an exoskeleton, wearing an exoskeleton reduced work metabolic heat production in both hot and cold environments. Metabolic rates slightly increased when wearing the exoskeleton under REST, but the effect was not significant.
2. LIFTING in TEMP was observed have a higher metabolic rate than COLD under both WEXO and EXO. Under REST, the metabolic rate in COLD was higher than TEMP, but it was not significant.
3. For the LIFTING task, using the exoskeleton in TEMP increased the MST, sweating and thermal sensation, thereby increasing the human thermal discomfort. However, in the COLD environment, the thermal comfort and sweating was significantly improved by wearing the exoskeleton.
4. For REST, the MST and thermal sensation in both environments were significantly improved by wearing an exoskeleton, whereas the improving effect of wearing an exoskeleton on thermal comfort was only observed in the cold environment.
5. In addition to the support function, the increase in local clothing insulation is one of the key factors affecting thermal responses. Wearing an exoskeleton causes a reduction of the “pumping effect” during work.

Although the exoskeleton can effectively reduce work intensity, the thermal discomfort of the user may hinder the positive effects of its use. Hot environments look very unsuitable for using exoskeletons. Therefore, it is necessary to carefully evaluate the thermal response in the design stage and develop appropriate usage strategies according to the temperature.

## Figures and Tables

**Table 1:** The environmental parameters tested in the experiments.

Tested Environmental Parameters	TEMP		COLD	
	Mean	SD	Mean	SD
Indoor air temperature (°C)	25	0.2	10.1	0.4
Mean radiant temperature (°C)	25.2	0.2	10.3	0.2
Air velocity (m/s)	<0.1		<0.1	
Relative humidity (%)	50	2.7	50	3.1

## Data Availability

The data presented in this study are available on request from the corresponding author. The data are not publicly available due to privacy.

## Appendix A

**Table A1:** Mean (SD) values of metabolic rate (W/m^2^) during the experimental sessions.

	WEXO				EXO			
	REST		LIFT		REST		LIFT	
	Mean	SD	Mean	SD	Mean	SD	Mean	SD
TEMP	68.08	13.90	313.49	32.31	68.08	16.61	225.53	26.53
COLD	77.14	12.49	273.15	45.80	74.32	16.02	196.73	33.39

**Table A2:** Mean (SD) values of mean skin temperature during the experimental sessions.

	WEXO				EXO			
	REST		LIFT		REST		LIFT	
	Mean (°C)	SD	Mean (°C)	SD	Mean (°C)	SD	Mean (°C)	SD
TEMP	33.07	0.59	33.87	0.53	33.48	0.53	34.13	0.80
COLD	31.22	0.70	29.95	1.06	31.78	0.73	30.37	1.15

**Table A3:** Mean (SD) values of thermal sensation vote during the experimental sessions.

	WEXO				EXO			
	REST		LIFT		REST		LIFT	
	Mean	SD	Mean	SD	Mean	SD	Mean	SD
TEMP	0.20	0.42	2.00	0.82	0.70	0.48	2.60	0.70
COLD	−2.00	0.67	−0.30	0.82	−1.20	0.42	1.20	0.92

**Table A4:** Mean (SD) values of thermal comfort vote during the experimental sessions.

	WEXO				EXO			
	REST		LIFT		REST		LIFT	
	Mean	SD	Mean	SD	Mean	SD	Mean	SD
TEMP	0.57	0.99	−0.86	0.98	0.21	0.64	−1.82	0.99
COLD	−0.86	0.98	−0.46	0.81	−0.02	0.10	−0.02	0.10

**Table A5:** Mean (SD) values of sweat feeling index during the experimental sessions.

	WEXO				EXO			
	REST		LIFT		REST		LIFT	
	Mean	SD	Mean	SD	Mean	SD	Mean	SD
TEMP	0.00	0.00	1.90	0.57	0.10	0.32	2.60	0.52
COLD	0.00	0.00	1.60	0.70	0.00	0.00	0.30	0.48
